# Cerebral flow variation at different intra-aortic balloon settings in cardiogenic shock

**DOI:** 10.1093/ehjci/jeac054

**Published:** 2022-03-14

**Authors:** Costanza Natalia Julia Colombo, Angelo Guglielmi, Francesco Mojoli, Guido Tavazzi

**Affiliations:** 1 Department of Anesthesia, Intensive Care and Pain Therapy, Fondazione Policlinico San Matteo Hospital Viale Golgi, 19 - 27100 - Pavia, Italy; 2 Department of Clinical, Surgical, Diagnostic and Pediatric Sciences, University of Pavia, Corso Strada Nuova, 65 - 27100 - Pavia, Italy

A 65-year-old lady was admitted due to cardiogenic shock related to acute decompensated heart failure refractory to medical therapy, requiring oro-tracheal tube and intra-aortic balloon pump (IABP) placement. In addition to echocardiography, transorbital transcranial colour Doppler (TCOD) was performed to assess cerebral flow pattern at different steps of IABP support; the figure shows TCOD and echocardiographic images, respectively in the upper and lower panel, during these different phases: IABP 1:1, first step (*Panel A*), IABP 1:2, second step (*Panel B*), and IABP switched off, third step (*Panel C*).

At the first step (*Panel A*), prevalent (augmented) diastolic peak flow velocity (systolic peak 62 cm/s, diastolic peak 132 cm/s) with interrupted flow in protosystole referring to the electrocardiogram was observed as compared to second (*Panel B*) and third step (*Panel C*). In the latter, normal flow pattern was resembled with lower diastolic velocity but continuous flow (systolic peak 95 cm/s, diastolic peak 44 cm/s). The IABP changes induced also aortic velocity time integral (first step: 9 cm/s; second step: 4 cm/s; and third step: 7 cm/s) and systemic blood pressure variations (first step: 115/81/65 mmHg; second step: 86/62/53 mmHg; and third step: 98/70/56 mmHg).

**Figure:**
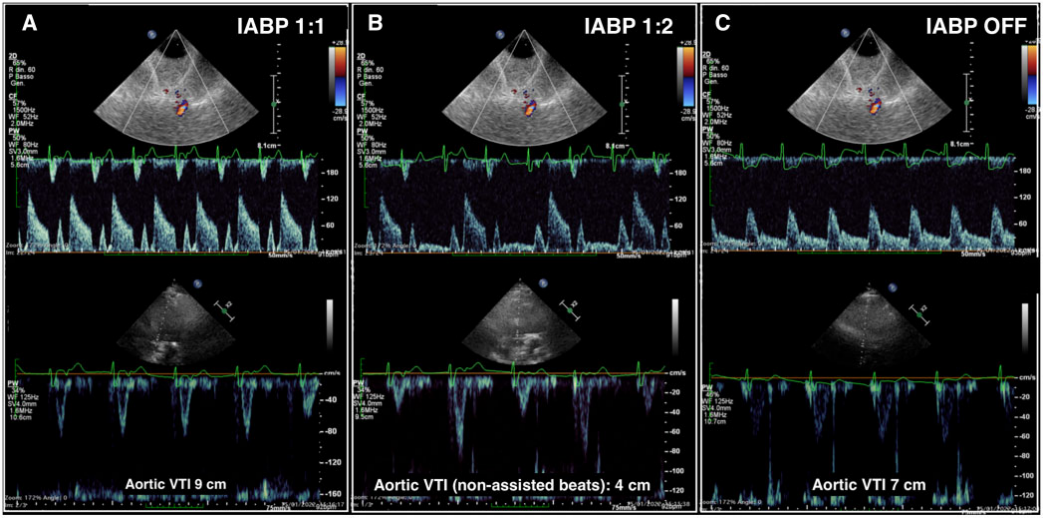


IABP balloon pump 1:1 resulted in an improvement in stroke volume generation which in turn led to better haemodynamic profile that coincided with a higher diastolic peak flow at TCOD. Whether higher diastolic peak flow but discontinuous is better than normal flow pattern (systolic > diastolic), although with lower velocities in term of cerebral perfusion, is still unknown.

